# Event and Comment

**Published:** 1922-05

**Authors:** 


### DENTAL REGISTER

THE MAGAZINE OF DENTISTRY

Vol. LXXVI MAY, 1922 No. 5

### EVENT AND COMMENT

**Is Dental Radiography Malpractice?** Can the art of radiography be considered as malpractice when it is as ordinarily used in the making of radiographic pictures?

We are printing herewith an editorial, "**So-Called Diagnosis**" from the *Pacific Dental Gazette* for April, which makes a strong arraignment of radiographers, as such, for the common practice of these people, because they overstep their legitimate functions as picture makers and give advice as to diagnostic symptoms of diseases, or of malpositions showing surgical or remedial treatments.

This problem has been under consideration for several years and its solution seems no nearer a satisfactory answer as to its ethics and legal rights and practices than when first advocated. It really seems necessary that our system of nomenclature will need to be revised, or some new rules of ethics be adopted for further guidance. Possibly some new amendments to our statutes will make the way clearer for a more explicit rendering of the rights and privileges of all concerned.

By common consent we have permitted the technical artisans of the prosthodontists and the dental hygienists to legally and traditionally consent to qualify in both of these respects, and they both now engage satisfactorily in most important health functions, and without criticism. Then why should not the radiographer become equally valuable and creditable in our general system of the health programme without being ostracised or debarred from this most necessary and useful occupation? Most good things can be misinterpreted and even abused, but these helpful and important adjuncts are at this time surely needed, and we can well afford to wait for the fullness of time and the adjustment of corrected methods and refining influences. The process of evolution will finally produce the proper end results. Read and ponder the editorial in the *Gazette* and impatiently wait for the ideal for which we anxiously aim:

The dental law of the State of California very explicitly determines the conditions under which certain properly qualified persons may engage in this state in the practice of dentistry. The law is very clear on the definition of dentistry, stating that within the meaning of the act any person shall be understood to be practicing dentistry who shall for a fee, salary or reward paid directly or indirectly, either to himself or to some other person, perform the operations outlined in the law or **make an examination of, with intent to perform or cause to be performed any operation in the human teeth or jaws.** It is perfectly clear, therefore, that holders of the license to practice dentistry in this state as issued by the Board of Dental Examiners are the only ones permitted by law to undertake the examination of the mouth and teeth and to dispense advice concerning these structures and organs, as well as to recommend the treatment to which they should be subjected. The examination of the mouth and all its contained organs and structures for the purpose of determining the existence of abnormal or pathological conditions and to outline the corrective measures—surgical, operative and prosthetic—which may be called for by the diagnostic findings is without question or exception one of the most difficult problems which may enlist the attention of the dental practitioner. Each patient presents such a complexity of circumstances and such diversified pathologic reactions that unless the diagnostician is by previous educational training and clinical experience in a position to avail himself of, and to co-ordinate the information securable from such subjects as anatomy and histology, physiology and pathology, chemistry and bacteriology, and in addition to this all the purely technical dental subjects, his results will be injurious and detrimental rather than beneficial to the future welfare of his patients. If dental diagnosis is even, when undertaken by those professionally qualified to do so, a task of such complexity, involving responsibilities of the highest order and calling for the dispensation of services ranking in importance above all other types of services which the dental profession may render, why then, we ask, should these duties be assumed by those who neither professionally nor in any other way have any vestige of right to intrude themselves into the professional field.

We have repeatedly pointed out in the past that dental diagnosis is not dependent upon radiography to the exclusion of other methods of obtaining accurate data concerning the condition of the teeth and their investing structures. The value of radiography in dental diagnosis is of the greatest significance; it is an indispensable diagnostic adjunct; it will assist in detecting lesions in their incipiency which in no other way could possibly be even suspected. In the hands of the properly qualified individual it is an instrument for good, the steadfast antagonist of misguided and misdirected curative efforts, an agency in the domain of prophylactic dentistry of consummate value. In the hands of the educationally and professionally unqualified its inherent virtues and potent possibilities for good as a diagnostic medium are almost entirely wiped out, and it then becomes a dangerous weapon, insiduously preventing the recovery of dental and general maladies or assisting in the development of new ones.

It has been said by those whose statements could not be reasonably contradicted that if a layman assumes the authority to interpret radiograms, by that same authority he should practice other branches of medicine and dentistry. Is the dental profession going to tolerate this deplorable condition of affairs indefinitely? Time will tell.

---

**Memorial Monument to Chapin A. Harris.** A substantial and most fitting memorial is to be erected in Baltimore, June 30, 1922, for the founder of dental education, Chapin A. Harris. The history of dentistry, as it is related to this country can hardly be mentioned without special attention to the part which Chapin A. Harris took in establishing modern dentistry in this country, if not in all the world. Everybody knows what Harris stood for and how he worked and strove to establish dentistry as a special branch of the healing art. We as a profession, will always honor him for what he did; but perhaps none will realize the great fact that he did his work absolutely without a personal motive for gain, or even for glory. It was in every sense a contribution to humanity, and was divinely inspired. The proposal to erect this monument is particularly appropriate and does us all credit. Some money will be needed, and a universal and liberal gift should quickly be forthcoming. Any gift will be accepted and gratefully appreciated by the Committee. Send contributions to Dr. W. G. Foster, North Howard St., Baltimore, Md.

